# Exploring the Neuroprotective Effects of Lithium in Ischemic Stroke: A literature review

**DOI:** 10.7150/ijms.88195

**Published:** 2024-01-01

**Authors:** Weihua Wang, Dunlin Lu, Youkui Shi, Yanqiang Wang

**Affiliations:** 1 Department of Emergency, Affiliated Hospital of Weifang Medical University, Weifang, Shandong 261031, P.R. China.; 2 Department of Neurology Ⅱ, Affiliated Hospital of Weifang Medical University, Weifang, Shandong 261031, P.R. China.

**Keywords:** Ischemic stroke, Neuroprotection, Lithium, Molecular mechanism, Mitochondrial dysfunction, Inflammatory marker

## Abstract

Ischemic stroke ranks among the foremost clinical causes of mortality and disability, instigating neuronal degeneration, fatalities, and various sequelae. While standard treatments, such as intravenous thrombolysis and endovascular thrombectomy, prove effective, they come with limitations. Hence, there is a compelling need to develop neuroprotective agents capable of improving the functional outcomes of the nervous system. Numerous preclinical studies have demonstrated that lithium can act in multiple molecular pathways, including glycogen synthase kinase 3(GSK-3), the Wnt signaling pathway, the mitogen-activated protein kinase (MAPK)/ extracellular signal-regulated kinase (ERK) signaling pathway, brain-derived neurotrophic factor (BDNF), mammalian target of rapamycin (mTOR), and glutamate receptors. Through these pathways, lithium has been shown to affect inflammation, autophagy, apoptosis, ferroptosis, excitotoxicity, and other pathological processes, thereby improving central nervous system (CNS) damage caused by ischemic stroke. Despite these promising preclinical findings, the number of clinical trials exploring lithium's efficacy remains limited. Additional trials are imperative to thoroughly ascertain the effectiveness and safety of lithium in clinical settings. This review delineates the mechanisms underpinning lithium's neuroprotective capabilities in the context of ischemic stroke. It elucidates the intricate interplay between these mechanisms and sheds light on the involvement of mitochondrial dysfunction and inflammatory markers in the pathophysiology of ischemic stroke. Furthermore, the review offers directions for future research, thereby advancing the understanding of the potential therapeutic utility of lithium and establishing a theoretical foundation for its clinical application.

## 1. Introduction

Stroke is defined as a neurological deficit resulting from acute focal injury to the central nervous system of vascular origin 1. According to the Global Burden of Diseases, Injuries, and Risk Factors Study (GBD) 2019, stroke remained the second leading cause of death and the third leading cause of disability globally 2. Stroke types are primarily classified as ischemic and hemorrhagic strokes, with diagnosis and differentiation based on clinical features and brain imaging 1. In 2019, ischemic stroke constituted 62.4% of all newly diagnosed stroke cases, while intracerebral hemorrhage constituted 27.9% of cases worldwide 2. Ischemic stroke, the predominant etiology among stroke types, accounts for about 87% of all cases 3.

Restoring blood flow (reperfusion) and preventing cell damage (neuroprotection) are two potential treatment strategies for ischemic stroke 4. To restore blood perfusion, thrombolytic therapy and endovascular therapy are available 4. Thrombolytic therapy is most effective when initiated within 4.5 hours of an acute stroke, given its narrow time window 5. Patients with stroke episodes beyond 4.5 hours, proximal artery occlusion, and contraindications to thrombolysis are not eligible for intravenous thrombolysis 6. In addition, intravenous tissue-type plasminogen activator (t-PA) carries a risk of symptomatic intracranial hemorrhage (sICH) 4, 7. Endovascular therapy proves effective for acute occlusion of large vessels 8, encompassing direct injection of t-PA into the artery (arterial thrombolysis) and mechanical thrombectomy 6. The clinical effect of arterial thrombolysis remains unclear. While thrombectomy has gained widespread use in clinical practice, it is associated with a higher rate of surgical complications 6, 9. Mechanical thrombectomy is recommended within 6 hours from symptom onset in patients with large vessel occlusion (LVO), either in combination with intravenous thrombolysis within 4.5 hours of symptom onset or as a standalone procedure between 4.5 hours and 6 hours of symptom onset 10.

Progressive neurodegeneration and loss of function caused by stroke affect the quality of life of patients after stroke. Most patients suffer from sequelae such as cognitive impairment, movement disorders, depression, swallowing disorders, and language disorders 11. Consequently, the quest for stroke neuroprotectors aimed at improving neurological outcomes has become a paramount focus, drawing attention from researchers worldwide.

Lithium was initially used for the treatment of urinary tract stones, but it later gained widespread acceptance for treating psychiatric disorders 12. Lithium possesses antiviral properties 13, 14. Preclinical evidence suggests its inhibitory effects extend to specific Coronaviridae viruses 15. Lithium demonstrates the capacity to extend lifespan in various animal models and humans 16. It proves beneficial for the cardiovascular system, renal function 17, bone metabolism, orthodontically induced root resorption (OIRR), and primordial follicle activation 18-20. Furthermore, lithium shows promise in addressing conditions such as diabetes (including type 2 diabetes and the diabetogenic effects of chronic corticotherapy 21), obesity, osteoporosis, and sarcopenia 19. Additionally, lithium exhibits effects on microorganisms 22. These interdisciplinary studies contribute to a more comprehensive understanding of lithium's potential applications.

**Figure 1:**
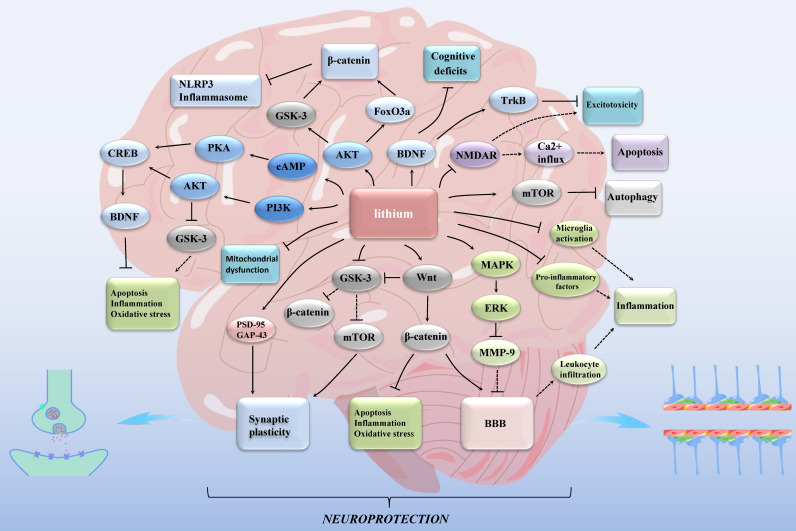
Lithium exerts neuroprotective effects in ischemic stroke through multiple mechanisms. GSK-3: glycogen synthase kinase 3; mTOR: mammalian target of rapamycin; MAPK: mitogen-activated protein kinase; ERK: extracellular signal- regulated kinase; MMP: matrix metalloproteinase; NMDAR: N-methyl-d-aspartate receptor; FoxO3a: forkhead box O3; NLRP3: nucleotide-binding oligomerization domain, leucine-rich repeat and pyrin domain-containing protein 3; cAMP: cyclic adenosine monophosphate; PKA: protein kinase A; PI3K: phosphatidylinositol 3-kinase; AKT: protein kinase B; CREB: cAMP-response element binding protein; BDNF: brain-derived neurotrophic factor; Trk: tropomyosin-related kinase; PSD-95: postsynaptic density protein 95; GAP-43: growth associated protein-43; BBB: blood-brain barrier. Lines with solid arrows represent stimulatory connections; lines with flattened ends represent inhibitory connections. Dashed lines represent pathways with reduced activity as a result of lithium treatment.

Lithium exhibits its effects through rodent models 12, in vitro cellular models 23, 24, and in vivo substitution models such as Eisenia fetida 25. Individuals with different genotypes may respond differently to lithium treatment 12, 26. While experimental models have demonstrated the impact of lithium on ischemic stroke 27, its mechanism remains incompletely understood 12. Furthermore, its clinical application is not well-established 27. In this review, our emphasis is on elucidating the neuroprotective role of lithium against ischemic stroke (Figure 1). Additionally, we explore the potential of targeting mitochondria and inflammatory markers as viable therapeutic avenues for stroke.

## 2. Molecular mechanism

### 2.1 GSK-3

Glycogen synthase kinase-3 (GSK-3), a serine/threonine kinase comprising α and β isoforms, represents a key target of lithium 28. In contrast to the typical behavior of many protein kinases, GSK-3 is inherently active in unstimulated cells and experiences inhibition upon stimulation 28. Its substrates often require an additional "trigger phosphorylation event," and GSK-3 demonstrates a broad spectrum of substrates 28, reflecting its involvement in diverse cellular processes, including cell proliferation and differentiation, cell cycle regulation, apoptosis, and autophagy 29, 30.

GSK-3 can be inhibited by lithium through direct binding to the adenosine triphosphate (ATP)-dependent magnesium-sensitive catalytic site of the enzyme. Additionally, lithium indirectly suppresses GSK-3 activity by promoting its serine phosphorylation through the phosphatidylinositol 3-kinase (PI3K)/protein kinase B (AKT), PI3K/protein kinase C (PKC), and cyclic adenosine monophosphate (cAMP)/protein kinase A (PKA) signaling pathways 31. Lithium enhances the serine phosphorylation of GSK-3 by disrupting the β-arrestin-2 (βArr2)-protein phosphatase 2A (PP2A)-AKT complex, which plays a role in dephosphorylating and deactivating AKT. Moreover, through the disinhibition of inhibitor-2 (I-2) inhibitory action on protein phosphatase-1 (PP-1), which is responsible for the dephosphorylation of GSK-3 at serine residues, lithium's direct inhibition of GSK-3 disrupts the auto-regulation of GSK-3 and further decreases its activity 31, 32.

Lithium inhibits IMPase to induce mTOR-independent autophagy. Simultaneously, it inhibits GSK-3β, which, in turn, reduces autophagy by activating mTOR 33, 34. The differential effects of lithium on autophagy may be attributed to varying therapeutic concentrations 35.

The GSK-3 signaling pathway regulates autophagy by modulating downstream signaling molecules in both mTORC1-dependent and mTORC1-independent manners 36. mTOR is a well-established major autophagy regulator, and GSK-3 interacts with mTORC1 to influence autophagy. On the one hand, GSK-3 inhibitors can down-regulate the expression of mammalian target of rapamycin complex (mTORC1), thereby inducing autophagy. On the other hand, mTORC1 regulates Foxk protein (Foxk1) through GSK-3, leading to autophagy inhibition 36, 37. In the context of autophagy regulation, most studies have indicated that GSK-3 acts as a positive regulator of mTORC1 28, 38, aligning with the earlier findings mentioned. This modulation of autophagy promotes the clearance of harmful substances favorable in treating neurological diseases 28. However, some studies have suggested GSK-3 as a negative regulator of mTOR 39.

GSK-3β, the most extensively researched isoform of GSK-3 40, is widely distributed throughout brain tissue 41. It becomes activated during cerebral ischemia, and its activation is associated with adverse effects on post-ischemic neuronal survival 41. The inhibition of GSK-3β plays a vital role in regulating processes such as inflammation 42, autophagy 43 (including mitophagy 44), apoptosis 31, oxidative stress 45, excitotoxicity 31, and pyroptosis 46, thereby facilitating neuroprotection. Furthermore, the post-stroke administration of GSK-3β inhibitors enhances cognitive recovery and can mitigate recombinant tissue plasminogen activator (rt-PA)-induced hemorrhagic conversion 47, 48. In the context of oxygen-glucose deprivation (OGD)-induced neuronal injury, GSK-3 inhibitors exhibit the potential to diminish the generation of mitochondrial reactive oxygen species (ROS), thus alleviating mitochondrial damage in neurons 31. Consequently, GSK-3 emerges as a promising candidate for a therapeutic target in ischemic stroke.

Following hypoxia-ischemia (HI) in neonatal rats, lithium chloride induces axonal repair by inhibiting GSK-3β to activate the mTORC1 signaling pathway 49. A recent study indicated that lithium chloride ameliorates ischemic brain injury and alleviates associated cognitive impairment by inhibiting nucleotide-binding oligomerization domain, leucine-rich repeat and nucleotide-binding oligomerization domain, leucine-rich repeat and pyrin domain-containing protein 3 (NLRP3) inflammasome activation through AKT/GSK-3β/β-catenin and AKT/forkhead box O3 (FoxO3a)/β-catenin pathways 12.

### 2.2 The Wnt signaling pathway

The Wnt signaling pathway is an intercellular signaling cascade activated by lipid-modifying proteins secreted by the Wnt family. The most basic form of the pathway consists of Wnt ligands from the secreting cell, their cognate receptors on the surface of the receiving cell, and signaling sensors within the receiving cell 50. Subsequent responses elicited by the Wnt signaling pathway play an essential role in a variety of physiological aspects of an organism 51. The Wnt signaling pathway is a complicated regulatory network consisting of two branches: canonical and non-canonical pathways 52. The canonical Wnt signaling pathway is also known as the Wnt/β-catenin signaling pathway. The Wnt/β-catenin signaling pathway is involved in vascular development 53 and BBB formation in the brain 54. In cerebrovascular endothelial cells, neurons, pericytes, astrocytes, microglia, and oligodendrocytes, the Wnt signaling pathway regulates cell survival and proliferation, even influencing their unique biological functions 55. Lithium chloride activates the Wnt signaling pathway to reduce infarct volume and alleviate neurological deficits 56.

Wnt proteins can also activate signaling pathways independent of Wnt/β-catenin, mainly including Wnt/ planar cell polarity (PCP) and Wnt/Ca2+ signaling pathways, collectively referred to as non-canonical Wnt signaling pathways. Previous studies have demonstrated some synergistic 57 and antagonistic effects 58, 59 between canonical and non-canonical Wnt signaling pathways. After ischemic stroke, both canonical and non-canonical Wnt signaling pathways are down-regulated. However, the blood-brain barrier (BBB) is protected by the up-regulation of Wnt/β-catenin signaling from the interference of the non-canonical Wnt signaling pathway 60.

The activation of the typical Wnt signaling pathways leads to the inactivation of GSK-3β, resulting in the stabilization of β-catenin and facilitating its translocation to the nucleus 61. The Wnt signaling pathways activate mTOR by inhibiting GSK-3 to regulate cell growth, independent of β-catenin-dependent transcription 39. Furthermore, the Wnt pathways inhibit mTOR by suppressing the GSK-3/AMP-activated protein kinase (AMPK) pathway and inducing autophagy in hippocampal neurons 62. Both of these mechanisms may be unrelated to β-catenin. However, the inhibition of GSK-3β activity by lithium chloride administration in the oxygen-glucose deprivation/reperfusion (OGD/R) model might increase the expression level of β-catenin 63, 39. This study may elucidate the discrepancy, suggesting that the accumulation of β-catenin after Wnt signaling activation and the regulation of mTOR through GSK-3 by Wnt may involve two distinct pathways.

### 2.3 The MAPK/ERK signaling pathway

The MAPK pathway, playing a pivotal role in cellular processes like proliferation, differentiation, and transformation, represents an evolutionarily conserved signaling cascade 64, 65. Comprising three primary subfamilies, including c-Jun-N-terminal kinase (JNK), p38, and ERK1/2 66, with ERK1/2 being integral to the classical pathway within the MAPK cascade 65. The MAPK/ERK1/2 signaling pathway is the most extensively studied among the MAPK pathways 67. Activation of all MAPK pathways occurs in cerebral ischemia, where JNK and p38 activation can be detrimental, and ERK1/2 activation may exhibit both beneficial and harmful effects 68. In light of these considerations, our focus now shifts to the neuroprotective role of the MAPK/ERK1/2 signaling pathway involved after ischemic stroke.

In experimental ischemic stroke studies, it is observed that lithium significantly improves the stability of the blood-brain barrier (BBB) through the activation of the MAPK/ERK1/2 pathway 69. Inhibiting the MAPK/ERK1/2 pathway increases neuronal apoptosis and significantly decreases cellular activity after cerebral infarction in rats 70. Additionally, both in vitro and in vivo experiments demonstrate that curcumin attenuates focal cerebral ischemia-reperfusion injury by positively regulating the MEK/ERK/cAMP-response element-binding protein (CREB) pathway 71.

The overexpression of ERK1/2 increased the infarct size and degree of neurological deficits in mice after transient middle cerebral artery occlusion (tMCAO). The stimulation of the MEK/ERK1/2 signaling proved detrimental to the functional outcome of ischemic stroke 72. Inhibiting the MEK/ERK1/2/CREB signaling pathway may enhance BDNF expression and exert neuroprotective effects, thereby improving neurobehavioral function in rats after middle cerebral artery occlusion/reperfusion (MCAO/R) 73. This result contradicts previous studies that suggested a neuroprotective effect of activating this pathway in ischemic stroke 69-71.

**Figure 2:**
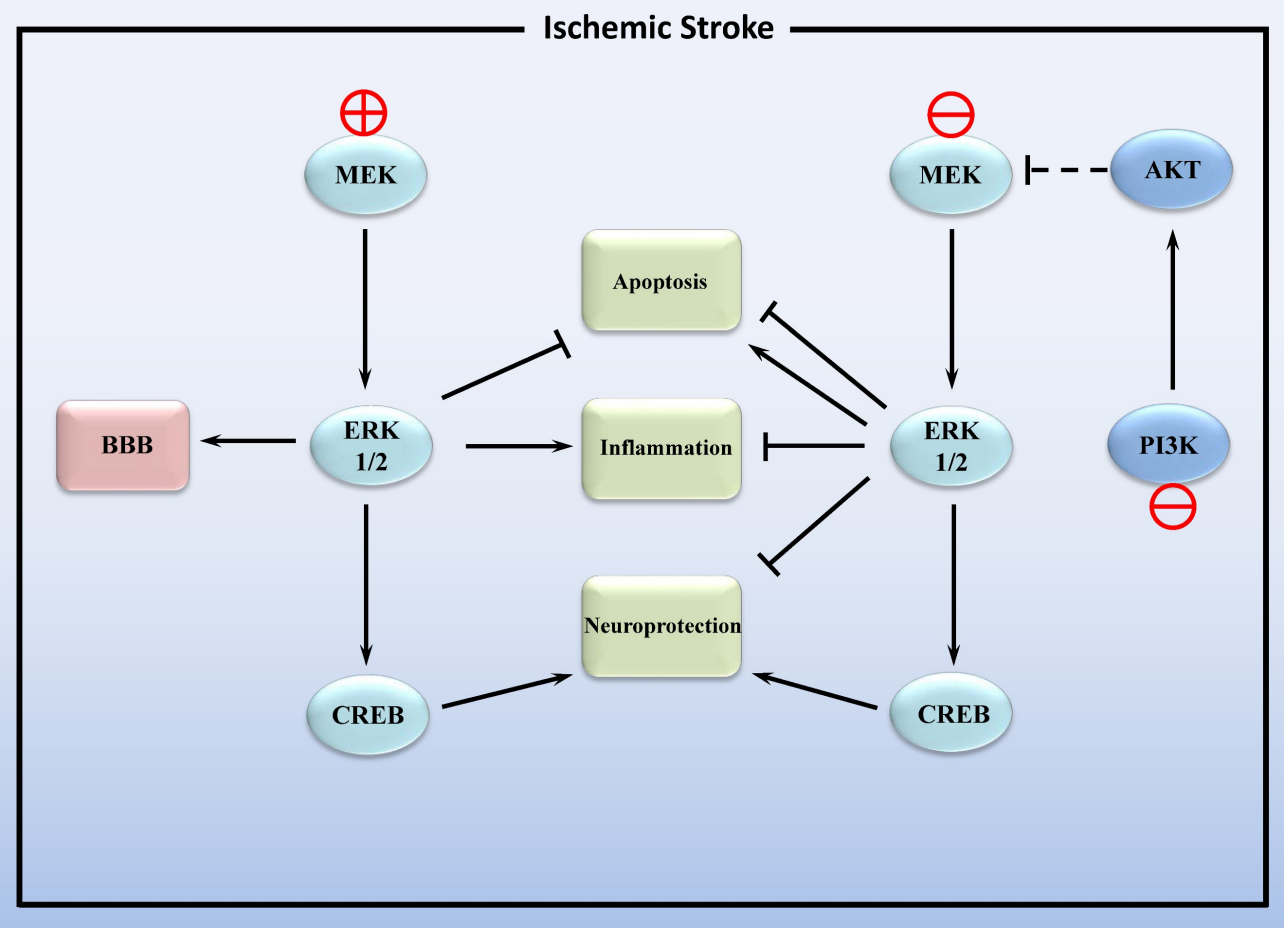
Activation and inhibition of the MEK/ERK1/2 signaling pathway in ischemic stroke. MEK: mitogen activated protein kinase; ERK1/2: extracellular signal- regulated kinase 1/2; CREB: cAMP -response element binding protein; BBB: blood-brain barrier. PI3K: phosphatidylinositol 3-kinase catalytic subunit type 3; AKT: protein kinase B. Lines with solid arrows represent stimulatory connections; lines with flattened ends represent inhibitory connections. Dashed lines represent pathways with reduced activity.

In conclusion, the MAPK/ERK1/2 pathway plays a dual role in cerebral ischemic stroke (Figure 2). On the one hand, activation of ERK1/2 inhibits apoptosis and exerts neuroprotective effects, yet also promotes inflammation. On the other hand, inhibition of ERK1/2 can alleviate post-ischemic inflammation and cellular injury by regulating downstream signaling pathways. This dual action may be related to whether the increase in ERK1/2 activity is due to injury such as stroke or caused by neuroprotective agents. Inhibition of the former-induced increase in endogenous ERK1/2 phosphorylation alleviated ischemic injury, and the increase in ERK1/2 phosphorylation induced by the latter reduces ischemic damage. The impact of additional factors, including cell surface receptor density and the extracellular matrix, may also result in distinct biological functions related to ERK1/2 activity 74, 75.

Intriguingly, a recent study demonstrated that lithium exerts its post-stroke neuroprotective activity through the PI3K/AKT pathway rather than the MEK/ERK pathway 77. It has been reported that there is crosstalk between the PI3K/AKT and MAPK/ERK pathways in cerebral ischemia-reperfusion (Figure 2). Following cerebral ischemia, the PI3K/AKT signaling pathway is activated, leading to the inactivation of MAPK/ERK1/2. During the reperfusion process, the PI3K/AKT signaling pathway is inhibited, thereby restoring the activity of the MAPK/ERK1/2 signaling pathway 78.

Hence, it is essential to delve deeper into the role of the MAPK/ERK signaling pathway in ischemic stroke.

### 2.4 BDNF

Neurotrophic factors constitute a diverse family of soluble molecules implicated in various neurological functions, including cell growth, differentiation, and plasticity 78. Among these factors, BDNF stands out as one of the extensively studied ones. BDNF, a factor with nerve growth-promoting activity, was first isolated and purified from the porcine brain in 1982 by Barde and others 79. Neurotrophic factors exert their effects by binding to two types of transmembrane receptors: tropomyosin-related kinase (Trk) family and p75 neurotrophin receptor (p75NTR) 80. Specifically, phosphorylated TrkB activates three major intracellular signaling pathways: the MAPK/ERK pathway, which influences cell growth and differentiation; the PI3K/AKT pathway, associated with cell survival; and the phospholipase C-γ (PLCγ)/Ca2+ pathway, which modulates synaptic plasticity 73, 81, 82. Elevated BDNF levels regulate the PI3K/AKT pathway through TrkB, contributing to a neuroprotective role in cerebral ischemia-reperfusion injury 83.

HI significantly reduces BDNF levels in various brain regions 84. HI induces BDNF/TrkB dysregulation, a phenomenon implicated in various neurological disorders, including ischemic stroke 85. BDNF, through the PI3K/AKT/mTOR signaling pathway, induces autophagy, providing neuroprotection against hypoxic injury in vitro 86. Experimental stroke models demonstrate that BDNF exerts neuroprotective effects, encompassing cognitive recovery promotion, neuroregeneration, anti-inflammatory and anti-neurotoxicity actions 85, 87. The majority of evidence supporting the neuroprotective effects of BDNF comes from preclinical trials, but its precise role in stroke patients remains unclear 88. Currently, there is insufficient evidence to consider BDNF as a biomarker for predicting functional outcomes in stroke 88. Nevertheless, during the acute phase of stroke, an inverse correlation exists between stroke severity and BDNF levels 88. Stroke patients prone to post-stroke depression (PSD) exhibit lower serum BDNF concentration levels in the early stroke phase compared to those without depression 89, 90. The BDNF Val66Met, a common single nucleotide polymorphism (SNP) in the human BDNF gene, may impact post-stroke recovery 91.

Lithium treatment alleviates cognitive deficits associated with cerebral perfusion injury by upregulating BDNF expression 92 and activates the BDNF/TrkB pathway in cortical neurons to prevent glutamate excitotoxicity 93. Both acute and chronic modes of lithium administration can elevate BDNF levels in the brain and reduce apoptotic levels, thereby achieving neuroprotective effects 94, 95. The regulation of multiple genes within specific brain regions by CREB is associated with various phenomena in neuropsychiatric disorders, and BDNF serves as a major downstream regulator of CREB 96. Lithium activates both the PI3K/AKT/CREB pathway and the cAMP/PKA/CREB pathway, leading to increased production of BDNF. This activation inhibits oxidative stress, inflammation, and apoptosis, achieving neuroprotective effects 97. Additionally, the inhibition of GSK-3β/CREB/BDNF 98 and MAPK/ERK/CREB/BDNF 73 signaling pathways can upregulate BDNF expression, thereby alleviating cerebral ischemia-reperfusion injury.

The activation of metabotropic glutamate receptor (mGluR) 2/3 through the application of mGluR2/3 agonists increases BDNF expression in the brains of newborn mice. Therefore, considering the interaction between neurotrophic factors and the mGlu2/3 receptor could be viewed as a potential mechanism involved in neuroprotection 84.

### 2.5 mTOR

mTOR, a serine/threonine kinase, holds significance as a member of the phosphoinositide 3-kinase-related kinase (PIKK) family 99. mTOR forms two distinct complexes, mTORC1, and mTORC2, through interactions with multiple chaperone proteins. The kinase activity of mTOR has been implicated in the pathogenesis of various diseases, including cancer and central nervous system disorders 100.

After the occurrence of cerebral ischemia, mTOR undergoes inhibition due to an insufficient energy supply, triggering autophagy 101. mTOR exerts a neuroprotective effect by affecting autophagy 101-103. Lithium chloride plays a crucial role in exerting its neuroprotective effects on ischemic stroke by activating the Wnt signaling pathways 54, 56 and inhibiting GSK-3 31. mTOR is regulated by both GSK-3 and the Wnt pathway 38, 39. The activation of mTOR by lithium chloride through the Wnt/GSK-3β pathway inhibits autophagy, alleviating ischemic brain damage 63. Furthermore, lithium chloride demonstrates the capacity to activate mTOR, restraining excessive autophagy and consequently ameliorating spatial cognitive deficits in murine models of cerebral ischemia-reperfusion 104. In the case of neonatal rats following hypoxic-ischemic injury, lithium chloride exerts its influence through the GSK-3β/mTORC1 signaling pathway, inducing axon repair 49.

It is noteworthy to recognize that lithium's influence on mTOR extends beyond ischemic stroke. Lithium demonstrates the ability to suppress autophagy through the Wnt/GSK-3/mTOR pathway in diabetic rat myocardial cells 105. Lithium facilitates physiological ventricular remodeling post-myocardial infarction by activating the PI3K/AKT/mTOR signaling pathway, suggesting potential implications for heart attack therapeutics. Additionally, lithium exhibits the capability to inhibit corticosteroid-induced chondrocyte autophagy 106, 107. Methylamphetamine (MA) possesses neurotoxic properties, and lithium treatment enhances the phosphorylation of Akt/GSK-3β/mTOR pathways, thereby mitigating the detrimental effects of MA on neuronal cells 108. Lithium treatment activates the AKT/mTOR pathway, leading to necrotic apoptosis and the demise of schwannoma cells, implying potential anti-tumor effects of lithium 109.

Ferroptosis, a form of cell death driven by iron-dependent lipid peroxidation, was identified as a distinct form of regulated cell death 110. Ferroptosis was first proposed in 2012 by Dixon in cancer research 111, and recent evidence suggests that ferroptosis is also present in the post-ischemic brain 112, 113. While it was once believed that ferroptosis remained impervious to other mechanisms, recent studies have contested this idea. The process of ferroptosis is not solely influenced by autophagy but is also affected by other factors, including inflammation 114, 115 and oxidative stress 116.

According to a recent report, ROS-induced autophagy promotes cellular ferroptosis 117. Given that ROS-induced autophagy and subsequent ferroptosis may contribute to myocardial injury after a heart attack, idebenone attenuates ferroptosis by inhibiting excessive autophagy through the ROS/AMPK/mTOR pathway to preserve cardiac function post-infarction 118. In trophoblast cells exposed to high concentrations of glucose and ferroptosis-inducing compounds, Sirtuin 3 (SIRT3) knockdown inhibits the AMPK/mTOR pathway and enhances glutathione peroxidase 4 (GPX4) levels to resist autophagy-dependent ferroptosis 119. Ferroptosis induced by the type 3 ferroptosis inducer (Fin56) in bladder cancer cells is also autophagy-dependent, and inhibiting autophagy attenuates the degradation of ferritin and GPX4. Although Fin56 may induce autophagy independent of mTOR, the ferroptosis triggered by Fin56 can be facilitated through autophagy mediated by mTOR inhibition 120.

Notably, mTOR and GPX4 exhibit interactions: (a) mTORC1 inhibition sensitizes cancer cells or tumors to ferroptosis and acts synergistically with ferroptosis inducers to inhibit tumor growth 121, (b) mTORC1 inhibition reduces GPX4 levels 121, 122 and (c) RSL3 blocks mTOR activation 121, 123.

Based on the available studies, it can be inferred that lithium holds therapeutic potential for ferroptosis. It is worth exploring whether lithium can modulate autophagy through mTOR to inhibit ferroptosis in ischemic stroke models.

### 2.6 Glutamate receptors

Glutamate receptors play a crucial role in physiological processes such as memory, learning, and synaptic plasticity. These receptors can be broadly classified into two types: ionotropic and metabotropic. In the mammalian brain, three ionotropic glutamate receptors exist: the N-methyl-d-aspartate receptor (NMDAR), the α-amino-3-hydroxy-5-methyl-4-isoxazole-propionic acid receptor (AMPAR), and the kainate receptor (KAR) 124. The conventional understanding of signal transduction by ionotropic glutamate receptors involves glutamate binding, which opens ion channels, allowing the passage of sodium, potassium, and calcium ions and generating excitatory signals 125. Metabotropic glutamate receptors (mGluRs) are G protein-coupled receptors 126.

#### 2.6.1 Ionic glutamate receptors

NMDAR are glutamate-gated ion channels widely expressed in the central nervous system and are crucial for neuronal communication 127. NMDARs form tetrameric complexes composed of two obligatory GluN1 subunits along with two GluN2 or GluN3 subunits, of which there are four (GluN2A-GluN2D) and two subtypes (GluN3A and GluN3B) respectively 128. GluN2A and GluN2B are the major subunits of functional NMDAR 129.

Recent studies have presented a paradox concerning the functional properties of NMDAR: only excessive activation of NMDAR leads to deleterious effects. In the context of ischemic brain injury, physiological activation of NMDAR is crucial for neuroplasticity and regeneration 127. Two hypotheses, the "NMDAR subtype" and "NMDAR location," have been proposed to elucidate the dual role of NMDAR in neuronal survival and death 130.

The "NMDAR subtype" hypothesis suggests that GluN2AR promotes neuronal survival while GluN2BR induces neuronal death. The specifics of the "NMDAR location" hypothesis remain controversial: one is that activation of synaptic NMDARs facilitates survival, while activation of extra-synaptic NMDARs leads to neuronal death; the other is that activation of synaptic or extra-synaptic NMDARs alone promotes neuronal survival, while co-activation of synaptic and extra-synaptic NMDARs is responsible for stroke injury. Co-activation is necessary to induce stroke injury. Both hypotheses remain to be further confirmed 126, 130.

NMDAR-mediated excitotoxicity can also cause different forms of neuronal death during cerebral ischemia, such as autophagy, apoptosis, ferroptosis, and parthanatos 131. The primary therapeutic approaches for managing excitotoxicity in ischemic stroke involve targeting glutamate, NMDAR, and downstream death signaling proteins 131. However, the clinical utility of NMDAR antagonists is restricted by the considerable side effects and relatively low efficiency. In contrast, the modulation of glutamatergic transmission through mGluRs to mitigate neurological damage may represent a safer and more effective therapeutic strategy 132.

And the neuroprotective effect of lithium is related to the inhibition of NMDAR-mediated calcium inward flow and downstream signaling 31. In addition, the synaptic protective effect of lithium is also mediated by activated NMDAR 133.

In the model of cerebral ischemia, lithium protects against excitotoxic damage by inhibiting the phosphorylation of NR2A and NR2B subunits and downregulating NMDAR 134, 135. However, some investigators have demonstrated through ex vivo experiments that lithium chloride can increase NMDAR expression by inhibiting GSK-3β 136.

NMDAR is related to the ability of learning and memory functions 137, 138. Prolonged lithium treatment impairs spatial memory function in rats 139, and NMDAR may contribute to the impact of lithium on inhibiting memory consolidation in rats 140. Generally, lithium demonstrates neuroprotective effects in pathological conditions. In the process of cerebral ischemia, lithium chloride can mitigate cognitive impairment caused by cerebral ischemia by inhibiting excessive autophagy, suppressing apoptosis, and increasing BDNF expression 104, 141.

#### 2.6.2 Metabotropic glutamate receptors

Based on sequence similarity, pharmacology, and intracellular signaling mechanisms, mGluRs are divided into group I, II, and III mGluRs. Group I mGluRs (mGluR1 and mGluR5) are associated with G proteins and coupled to phospholipase C (PLC). Group II (mGluR2 and mGluR3) and III mGluRs (mGluR4, mGluR6, mGluR7, and mGluR8) are associated with G proteins and negatively coupled to adenylate cyclase, and they are distributed in various brain regions 142, 143.

The regulation of mGluRs plays a role in various neuropsychiatric diseases, including Alzheimer's disease (AD) and Huntington's disease (HD) 144, 145. The subsequent discussion delves into their roles in cerebral ischemia and investigates the potential involvement of lithium as a neuroprotective agent.

##### Group I mGluRs

mGluR1 antagonists exhibit neuroprotective effects in ischemic stroke, potentially linked to the phosphorylation of the NMDAR subunit NR2A 146, 147. Notably, the neuroprotective efficacy of mGluR1 antagonists is comparatively weaker than that of mGluR5 antagonists 148.

The selective mGluR5 antagonist MPEP and agonist CHPG exhibit varying degrees of efficacy in alleviating ischemic brain injury in rat models 149, 150. However, in the focal cerebral ischemia model induced by endothelin-1, the mGluR5 agonist CHPG does not enhance brain function 151. The influence of mGluR5 on NMDAR function, including the inhibition of NR2A subunit phosphorylation, may be implicated in the neuronal cell death process in the ischemic stroke model 152, 153. These findings underscore the controversy surrounding the role of mGluR5 in ischemic stroke, emphasizing the need for further research and clarification in this area.

##### Group II mGluRs

A study has demonstrated that pretreatment with agonists of mGluR2/3 reduces apoptosis levels and provides neuroprotection against brain injury induced by cerebral ischemia in neonatal rats 126. However, another study adds evidence that the neuroprotection of mGluR2/3 agonists is mediated through the activation of mGluR3, not mGluR2 154. The selective mGluR2 negative allosteric modulators might alleviate ischemic neuronal death 155. Hereditary deletion of mGluR2 in a model of transient ischemia exerts neuroprotective effects 156. Notably, mGluRs and BDNF interact in the cerebral cortex. The gene expression of Group II mGluRs is negatively regulated by BDNF, whereas activation of group II mGluRs positively regulates BDNF expression levels 157. In a neonatal rat model of cerebral ischemia, group II mGluR agonists also upregulate BDNF levels, raising the possibility that crosstalk between mGluR and BDNF could offer new therapeutic targets for ischemic stroke 84. mGluR2/3 blockade induces behavioral deficits in mice, and clinically used antipsychotics such as lithium reverse the effects of mGluR2/3 antagonists 158. Enhanced mGluR-dependent long-term depression (mGluR-LTD) at the Schaffer lateral branch of hippocampal CA1 pyramidal synapses is a feature of a mouse model of fragile X syndrome 159. Both lithium and the group II mGluR antagonists treatment ameliorate enhanced mGluR-LTD in fragile X mice, a potential therapeutic agent for fragile X syndrome 159. Whether acting in ischemic stroke models or not, studies in which lithium directly affects mGluR2/3 are limited and deserve further exploration.

##### Group III mGluRs

Earlier studies indicated increased mRNA levels of mGluR4 in the hippocampus and parietal cortex after transient ischemia 160, 161. To investigate the role of mGluR4 in ischemic stroke, researchers utilized the middle cerebral artery occlusion (MCAO) model and the endothelin-1(Et-1) model of transient focal ischemia. They discovered that mGluR4 enhancer PHCCC administration reduced infarct size and improved sensorimotor function. In contrast, mice lacking mGluR4 exhibited greater brain damage than their wild counterparts from the same litter 162.

The group III mGluR agonist ACPT-I exerts neuroprotective effects in ischemic stroke and improves post-ischemic gait impairment 163. ACPT-I exerts similar effects in spontaneously hypertensive rats (SHR) after MCAO/R 132, 164.

mGluR6 is not expressed in the brain but only in the retina 165. mGluR7 activation may protect mouse neuronal cells from ischemic injury 166.

The role of lithium on metabotropic glutamate receptors is also worthy of consideration. Gq/PLC signaling is the target of lithium effects, and Gq/PLC-linked receptors include muscarinic receptors M1, 3, and 5, glutamatergic receptors mGluR1 and 5, adrenergic α1 receptors, and 5HT receptor subtype 2a-c 167.

Chronic lithium treatment of hippocampal neurons 168 and cortical neurons 169 resulted in a decrease in cell surface mGluR5 expression and a reduction in mGluR5-mediated intracellular calcium ion levels, while acute lithium treatment yielded null results. This observation may help explain the therapeutic effect of lithium in bipolar affective disorder. Altered excitatory/inhibitory synaptic transmission is also found in ischemic stroke 170. Multiple in vitro and in vivo experiments have demonstrated that lithium can play a role in ischemic stroke models 25. Lithium may benefit in improving clinical outcomes for stroke patients 25. Therefore, it is worthwhile to investigate whether lithium can exert a neuroprotective role in ischemic stroke models through mGluR5-mediated effects.

## 3. Mitochondrial dysfunction

Mitochondrial dysfunction has been identified as one of the main mechanisms involved in cerebral ischemic injury. The relationship between mitochondrial dysfunction and ischemic stroke has been studied more in recent years 171. Mitochondria are two-membrane-encapsulated organelles present in most eukaryotes 172. The primary role of mitochondria is to provide energy to the cell in the form of ATP through oxidative phosphorylation via the mitochondrial electron transport chain (ETC). Physiologically, about 90% of cellular energy is provided by mitochondria through the ETC 173.

Cerebral ischemia disrupts the ultrastructure of mitochondria, leading to mitochondrial dysfunction. In this process, the impairment of the mitochondrial electron transport chain initiates, leading to a cessation of ATP production. The diminished ATP production further disrupts ion transport. As ischemia advances, ATP levels continue to decrease, culminating in the accumulation of mitochondrial ROS and calcium ions. This cascade of events ultimately results in the opening of the mitochondrial permeability transition pore (mPTP) 174. mPTP opening has a significant effect on mitochondrial impairment 175, and it allows various solutes and water to enter the mitochondrial matrix, leading to the rupture of the outer membrane and swelling of the inner membrane, ultimately resulting in necrotic cell death 176. Endogenous apoptosis also involves the opening of the mPTP and the release of cytochrome c (cyt c).

The NLRP3 inflammasome plays a role in the pathological process of ischemic stroke. Following mitochondrial dysfunction, the release of ROS, mitochondrial DNA (mtDNA) damage, increased cardiolipin levels, and calcium influx into the cytoplasm are also involved in activating the NLRP3 inflammasome 177.

The mitochondrial quality control system, which includes mitochondrial dynamics (fission and fusion) and mitochondrial autophagy, is responsible for maintaining mitochondrial homeostasis, improving cellular function, and mitigating post-stroke brain damage 178.

Mitochondrial fusion involves the merging of two mitochondria into a single entity, with Mitofusin1 (Mfn1) and Mfn2 mediating outer mitochondrial membrane (OMM) fusion, while optic atrophy protein 1 (Opa1) mediates inner mitochondrial membrane (IMM) fusion. Mitochondrial fission involves the division of a mitochondrion into two smaller mitochondria, with a crucial role played by dynamin-related GTPase 1 (Drp1) 179.

Mitochondrial autophagy, a form of macroautophagy, is a selective process for removing damaged or unwanted mitochondria through autophagy. Diverse molecular mechanisms of mitophagy have been identified, including the phosphatase and tensin homolog-induced kinase 1 (PINK1)-Parkin pathway, the BCL2 and adenovirus E1B19-kDa-interacting protein 3 (BNIP3)/NIX pathway, the FUN14 domain-containing 1 (FUNDC1) pathway, and the cardiolipin pathway 180. Following a stroke, mitochondrial autophagy is inhibited 181. Proper mitochondrial autophagy plays a neuroprotective role by eliminating damaged mitochondria. However, excessive mitochondrial autophagy can lead to the over-degradation of intracellular components. The dual role of mitochondrial autophagy is influenced by various factors, including cell type, stroke progression, and variations in upstream stimulants 171.

Interventions such as estrogen replacement therapy, fission inhibitors, fusion activators, and modulation of mitochondrial autophagy can ameliorate mitochondrial dysfunction induced by cerebral ischemia. Consequently, developing pharmaceutical agents targeting mitochondria holds promise for advancing stroke treatment 171, 182.

Lithium inhibits the release of mitochondrial cytochrome C and apoptotic factors, mitigating brain injury in the HI model 31. Low concentrations of rotenone, a mitochondrial complex I inhibitor, induce mitochondrial dysfunction in cultured neurons in vitro. Lithium has shown potential in normalizing mitochondrial respiration and alleviating neuronal mitochondrial dysfunction by enhancing autophagy and modulating mitochondrial complex I activity 183. The respiration of mitochondria isolated from the ischemic brain is inhibited, likely attributable to damage in the mitochondrial respiratory chain induced by ischemia. In contrast, respiratory inhibition is alleviated in mitochondria from ischemic brains treated with lithium chloride 184. Further, in vivo experiments are needed to confirm the impact of lithium on mitochondrial dysfunction in ischemic stroke models.

## 4. Inflammatory markers

Inflammation plays a pivotal role in the pathogenesis of ischemic stroke. Ischemia triggers oxidative stress and excitotoxicity, leading to the activation of glial cells, which in turn secrete cytokines and matrix metalloproteinases (MMPs). Pro-inflammatory factors upregulate the expression of adhesion molecules on brain endothelial cells, facilitating the adhesion and infiltration of blood-borne leukocytes into the ischemic brain 185. Furthermore, dying or dead cells activate the innate immune system by releasing various danger-associated molecular patterns (DAMP). Activated immune cells further stimulate glial cells by secreting pro-inflammatory factors. These pathological events lead to neuronal death and exacerbate damage in the ischemic brain 185.

Post-stroke inflammation plays a dual role in the pathophysiology of ischemic stroke. In addition to pro-inflammatory factors contributing to post-stroke brain damage, anti-inflammatory factors and cells play a role in tissue and functional recovery after cerebral ischemia 68, 186.

Potential inflammatory markers can be classified into brain-specific, non-specific, and vascular markers, holding promise for stroke diagnosis, treatment response, and patient outcomes 186. Brain-specific inflammatory markers primarily encompass thiobarbituric acid reactive substances (TBARs), malondialdehyde (MDA), neuron-specific enolase (NSE), and heart-type fatty acid-binding protein (H-FABP). TBARs and MDA have shown predictive value for poor neurological outcomes, while NSE and H-FABP exhibit early elevation during cerebral ischemic events. Nevertheless, it is crucial to recognize that these markers possess limitations and are not currently employed in clinical practice 186. Vascular markers predominantly encompass d-dimer, intracellular adhesion molecule (ICAM)-1, vascular cell adhesion molecule (VCAM)-1, and E-selectin. Existing evidence implies the potential of these markers to predict post-ischemic functional outcomes. However, further confirmation is required through additional studies in the field of research 186. Non-specific inflammatory markers have been extensively investigated, mainly including C-reactive protein (CRP), interleukin (IL)-1β, IL-6, IL-10, and MMP-9 186.

CRP, an acute-phase protein, plays a role in activating several inflammatory mechanisms 187. Studies have identified inflammatory markers like CRP, IL-6, and IL-33 as predictors of initial stroke risk and post-stroke prognosis 188. Low levels of the anti-inflammatory cytokine IL-10 may predict an increased risk of stroke. Pre-clinical research has suggested that IL-10-deficient mice exhibit larger lesion sizes following MCAO 68.

Some immune cells can also serve as inflammatory markers for predicting stroke outcomes. One study has elucidated the association between inflammatory markers and four outcomes, including mortality and three primary indicators defined by the National Institutes of Health Stroke Scale (NIHSS), Modified Rankin Scale (mRS), and Barthel Index (BI) scores for functional outcomes. In this context, inflammatory markers such as neutrophils, lymphocytes, CRP, and IL-6 play an important role in predicting stroke severity as per the NIHSS, disability as per the mRS, poor outcomes according to the BI, and overall mortality 189.

Cognitive impairment affects approximately half of stroke survivors. Higher concentrations of plasma inflammatory markers are associated with cognitive impairment up to 36 months after stroke 190. In a study involving ischemic stroke patients, baseline neuropsychological assessments were conducted three months post-stroke, with annual psychological assessments over the next five years. The finding revealed that elevated IL-8 was independently associated with baseline cognitive impairment after ischemic stroke, while elevated IL-12 was linked to long-term cognitive decline 191. Inflammatory markers (IL-6 and CRP) are associated with post-stroke cognitive impairment 192.

Post-stroke depression (PSD) emerges as the predominant psychological consequence among stroke patients 193. Stroke-induced inflammatory responses are closely associated with the development of PSD 193. Inflammatory markers can predict PSD, with elevated CRP levels during the acute phase indicating an increased risk 193. IL-18 may also be involved in the development of PSD 194. Plasma fibrinogen level upon admission can also serve as an inflammatory marker for predicting PSD 193.

Beyond their diagnostic and prognostic roles in stroke, some inflammatory markers show promise as targets for stroke treatment. Inhibiting tumor necrosis factor (TNF)-α, MMP-9, and ICAM-1 may alleviate ischemic brain damage 187.

Some inflammatory pathologies may influence inflammatory marker levels. Identifying inflammatory markers presents a formidable challenge. Definitive markers meeting clinical prediction and treatment needs for ischemic stroke have not yet been established 187, 195. Further research is warranted to explore the potential utility of inflammatory markers for predicting functional outcomes and as targets for treating ischemic stroke.

Lithium treatment reduces microglia activation and the expression of pro-inflammatory factors following HI, promoting neuronal cell survival 196, 197. Lithium activates the MAPK/ERK pathway, which inhibits MMP-9, leading to enhanced BBB stability and reduced leukocyte infiltration in the ischemic brain 197. Co-administration of lithium and rutin reduces the expression of pro-inflammatory factors in a cerebral ischemia-reperfusion model 198. Additionally, lithium inhibits the activation of NLRP3 inflammasome, mitigating cerebral ischemic injury 12.

## 5. Summary and outlook

The mechanism of action of lithium in ischemic stroke has been extensively studied, but its precise mechanism remains a subject of controversy. Existing studies have demonstrated lithium can affect the treatment of some psychiatric disorders. This review summarizes that lithium exerts neuroprotective effects on ischemic stroke models through multiple pathways. However, the potential role of lithium in post-stroke recovery has not been thoroughly tested in humans. The issues raised in the review may be worth further investigation:

5.1 The MAPK/ERK signaling pathway is activated after ischemia-reperfusion. However, current studies suggest that the effect of activating this pathway on stroke remains elusive.

5.2 The concept of ferroptosis is relatively recent, and exploring its association with ischemic stroke may emerge as a future research direction. Within this realm, the impact of lithium on ferroptosis warrants additional investigation.

5.3 Current research indicates that scholars globally have frequently explored ionotropic glutamate receptors in ischemic stroke. The impact of mGluRs on ischemic stroke primarily occurs through their metabotropic modulators. Further investigation is needed to determine whether drugs targeting ionotropic glutamate receptors can also influence mGluRs.

5.4 The clinical application of inflammatory markers as predictive factors for ischemic stroke requires further experimental evidence. Nonetheless, this also offers new possibilities in treating ischemic stroke, which merits further exploration.

## Abbreviations

GSK-3: glycogen synthase kinase 3
MAPK: mitogen-activated protein kinase
ERK: extracellular signal-regulated kinase
BDNF: brain-derived neurotrophic factor
mTOR: mammalian target of rapamycin
CNS: central nervous system
GBD: Global Burden of Diseases, Injuries, and Risk Factors Study
t-PA: tissue-type plasminogen activator
sICH: symptomatic intracranial hemorrhage
LVO: large vessel occlusion
OIRR: orthodontically induced root resorption
ATP: adenosine triphosphate
PI3K: phosphatidylinositol 3-kinase
AKT: protein kinase B
PKC: protein kinase C
cAMP: cyclic adenosine monophosphate
PKA: protein kinase A
βArr2: βArr2β-arrestin-2
PP2A: protein phosphatase 2A
I-2: inhibitor-2
PP-1: protein phosphatase-1
mTORC: mammalian target of rapamycin complex
Foxk1: Foxk protein 1
OGD: oxygen-glucose deprivation
ROS: reactive oxygen species
HI: hypoxia-ischemia
rt-PA: recombinant tissue plasminogen activator
NLRP3: nucleotide-binding oligomerization domain, leucine-rich repeat and pyrin domain-containing protein 3
FoxO3a: forkhead box O3
PCP: planar cell polarity
BBB: blood-brain barrier
AMPK: AMP-activated protein kinase
OGD/R: oxygen-glucose deprivation/reoxygenation
JNK: c-Jun-N-terminal kinase
CREB: cAMP-response element binding protein
tMCAO: transient middle cerebral artery occlusion
MCAO/R: middle cerebral artery occlusion/reperfusion
Trk: tropomyosin-related kinase
PLCγ: phospholipase C-γ
p75NTR: p75 neurotrophin receptor
SNP: single nucleotide polymorphism
PSD-95: postsynaptic density protein 95
GAP-43: growth associated protein-43
mGluR: metabotropic glutamate receptor
PIKK: phosphoinositide 3-kinase-related kinase
SIRT3: Sirtuin 3
GPX4: glutathione peroxidase 4
Fin56: type 3 ferroptosis inducer
NMDAR: N-methyl-d-aspartate receptor
AMPA receptor: α-amino-3-hydroxy-5-methyl-4-isoxazole-propionic acid receptor
KA receptor: kainate receptor
mGluRs: metabotropic glutamate receptors
PLC: phospholipase C
AD: Alzheimer's disease
HD: Huntington's disease
mGluR-LTD: mGluR-dependent long-term depression
MCAO: middle cerebral artery occlusion
Et-1: endothelin-1
SHR: spontaneously hypertensive rats
ETC: electron transport chain
mPTP: mitochondrial permeability transition pore
cyt c: cytochrome c
mtDNA: mitochondrial DNA
Mfn1: mitofusin1
OMM: outer mitochondrial membrane
Opa1: optic atrophy protein 1
IMM: inner mitochondrial membrane
Drp1: dynamin-related GTPase 1
PINK1: phosphatase and tensin homolog-induced kinase 1
BNIP3: BCL2 and adenovirus E1B19-kDa-interacting protein 3
FUNDC1: FUN14 domain-containing 1
MMP: matrix metalloproteinase
DAMP: danger-associated molecular patterns
TBARs: thiobarbituric acid reactive substances
MDA: malondialdehyde
NSE: neuron-specific enolase
H-FABP: heart-type fatty acid-binding protein
ICAM: intracellular adhesion molecule
CRP: C-reactive protein
IL: interleukin
NIHSS: National Institutes of Health Stroke Scale
mRS: Modified Rankin Scale
BI: Barthel Index
PSD: post-stroke depression
TNF: tumour necrosis factor
